# Title II: Improving Grants for States and Community Programs on Aging

**DOI:** 10.1093/geroni/igaa057.2519

**Published:** 2020-12-16

**Authors:** Jamie Kuhne

**Affiliations:** Department of Veterans Affairs, Worthington, Ohio, United States

## Abstract

There are several noteworthy changes to the Improving Grants for States and Community Programs on Aging’s portion of the OAA. The reauthorization adds language requiring data collection on the needs of older adults and specifying additional populations on whom to focus outreach efforts, such as survivors of the Holocaust. The reauthorization also expands what States may fund with Supportive Services grants, adding screening for social isolation and traumatic brain injuries. The Act goes on extend Stated the option of funding programs to address both of these issues. The Act also requires the Assistant Secretary to study the supply and demand of home delivered and congregant meals and make recommendations to address the gap. Finally, there is expansion of the section on Caregiving, including a definition of caregiver assessment and the removal of a limit on funds States can use on support services to family caregivers.

